# Towards Engineering Novel PE-Based Immunotoxins by Targeting Them to the Nucleus

**DOI:** 10.3390/toxins8110321

**Published:** 2016-11-10

**Authors:** Marta Borowiec, Michal Gorzkiewicz, Joanna Grzesik, Aurelia Walczak-Drzewiecka, Anna Salkowska, Ewelina Rodakowska, Kamil Steczkiewicz, Leszek Rychlewski, Jaroslaw Dastych, Krzysztof Ginalski

**Affiliations:** 1 Laboratory of Bioinformatics and Systems Biology, Centre of New Technologies, University of Warsaw, Zwirki i Wigury 93, Warsaw 02-089, Poland; m.borowiec@cent.uw.edu.pl (M.B.); j.grzesik@cent.uw.edu.pl (J.G.); k.steczkiewicz@cent.uw.edu.pl (K.S.); 2 Laboratory of Cellular Immunology, Institute of Medical Biology, Polish Academy of Sciences, Lodowa 106, Lodz 93-232, Poland; mgorzkiewicz@cbm.pan.pl (M.G.); adrzewiecka@cbm.pan.pl (A.W.-D.); asalkowska@cbm.pan.pl (A.S.); jdastych@cbm.pan.pl (J.D.); 3 BioInfoBank Institute, Sw. Marcin 80/82 r.355, Poznan 61-809, Poland; ewelinarodakowska@bioinfo.pl (E.R.); leszek@bioinfo.pl (L.R.)

**Keywords:** Exotoxin A, nuclear localization signal, cytotoxicity, nuclease, cancer

## Abstract

Exotoxin A (PE) from *Pseudomonas aeruginosa* is a bacterial ADP-ribosyltransferase, which can permanently inhibit translation in the attacked cells. Consequently, this toxin is frequently used in immunotoxins for targeted cancer therapies. In this study, we propose a novel modification to PE by incorporating the NLS sequence at its *C*-terminus, to make it a selective agent against fast-proliferating cancer cells, as a nucleus-accumulated toxin should be separated from its natural substrate (eEF2) in slowly dividing cells. Here, we report the cytotoxic activity and selected biochemical properties of newly designed PE mutein using two cellular models: A549 and HepG2. We also present a newly developed protocol for efficient purification of recombinant PE and its muteins with very high purity and activity. We found that furin cleavage is not critical for the activity of PE in the analyzed cell lines. Surprisingly, we observed increased toxicity of the toxin accumulated in the nucleus. This might be explained by unexpected nuclease activity of PE and its potential ability to cleave chromosomal DNA, which seems to be a putative alternative intoxication mechanism. Further experimental investigations should address this newly detected activity to identify catalytic residues and elucidate the molecular mechanism responsible for this action.

## 1. Introduction

Exotoxin A (PE) is an ADP-ribosyltransferase produced by gram-negative bacterium *Pseudomonas aeruginosa*. It transfers ADP-ribosyl group from NAD+ to diphthamide residue of the eukaryotic EF2 elongation factor [1,2], thus permanently inhibiting translation in the attacked cell. Like many bacterial toxins, PE retains the “A-B” protein domain architecture composed of catalytic (A) and binding (B) domains [3,4]. The latter comprises a receptor domain (Ia, residues 1–252; Ib, residues 365–404) and the inserted translocation domain (II, amino acids 253–364). The receptor domain recognizes and binds to the alpha 2-macroglobulin receptor exposed on the cell surface (LRP receptor) [5,6], while the translocation domain is believed to participate in protein release from endocytotic vesicles to the cytoplasm after internalization, yet its detailed role remains unclear [2]. The PE catalytic domain is located *C*-terminally (III, residues 405–613) and performs ADP-ribosylation. The full-length toxin contains two sequence motifs critical for determination of its cellular trafficking and intoxication pathway: the *C*-terminal ^609^REDLK sequence important for retention from the endoplasmic reticulum [7], and the furin cleavage ^276^RQPR motif (located at the *C*-terminus of domain II) [8], which is recognized by ubiquitous furin protease. Furin seems to play a significant role in PE activation by cleaving away almost the whole domain B from the toxin (cut between residues R279 and G280) [9,10].

The natural potency of PE to cause cell death makes it a promising candidate for use in anticancer immunotoxins. These chimeric proteins are usually made of a modified antibody or antibody fragment, attached to a fragment of a toxin [11]. The “A-B” modular domain architecture of PE is convenient for engineering toxin targeting specificity by exchanging domain B for a specific antibody, which recognizes receptors exposed on the surface of targeted cancer cells, allowing the toxic agent (domain A) to be delivered to the cells of interest [12]. Therefore, the PE and closely related Diphtheria toxin (DT) are the most commonly tested toxins thus far, and numerous PE- and DT-based immunotoxins are currently subjected to various phases of clinical trials [13,14].

A number of drawbacks has been observed for the tested immunotoxins: lack of specificity, high immunogenicity, vascular leak syndrome (VLS) or hepatogenicity being the most deleterious [15]. The attempts to reduce the side effects focus mostly on: (i) improving the antibody specificity for antigens of interest and removing the cross-reactivity with others; and (ii) increasing toxicity of the effector moiety and thus lowering the administered dose of immunotoxin [16]. Another approach, which is proposed in this work, is based on lowering nonspecific toxicity of potential immunotoxins by reducing the access of the toxin to its natural substrate in slowly dividing cells and changing it to a cancer-selective compound without interfering with its cytotoxic potency.

One of the most remarkable hallmarks of the cancer cells is their proliferative capacity [17]. Therefore, in order to affect intensively dividing cancer cells, we propose a new strategy based on attaching the nuclear localization sequence (NLS) to the toxin and trapping it within the nucleus [18,19]. In non-dividing cells, nuclear membrane maintains its integrity and effectively separates the nuclear interior from the cytoplasm, whilst, in dividing cells, a periodic disintegration of nuclear membrane is observed. Since NLS targets the toxin to the nucleus, the cytoplasmic protein synthesis machinery should be protected from the unintended activity of the toxin until the cell division. Consequently, as long as the cell is in an unproliferative state, the access of the modified toxin to its natural substrate (eEF2) is limited. In a proliferating cell, in prometaphase, the nuclear envelope disruption would lead to the release of the toxin into cytosol, hence promoting cell death. This newly proposed mechanism should make the toxin active mainly against highly proliferating cancer cells.

The idea of attacking only fast proliferating cells was already exploited in a traditional cytotoxic chemotherapy which works primarily through the inhibition of cell division [20] with paclitaxel, cisplatin, doxorubicin and many others being frequently used in this type of treatment [21]. In addition to cancer cells, other rapidly dividing cells (e.g., hair, gastrointestinal epithelium, and bone marrow) are affected by these drugs, which causes numerous side effects (e.g., neutropenia, cytopenia, anemia, hair loss, skin itch, nausea, vomiting or diarrhea, and damage to liver, kidney and bone marrow) [22,23]. However, potential immunotoxins combining selective toxin, proposed in this work, with highly specific antibodies should be devoid of nonspecific toxicity.

Here, we report the cytotoxic activity and selected biochemical properties of a newly designed recombinant PE mutein enriched with NLS motif. We also present a novel protocol for PE purification developed in this study that allows obtaining highly active recombinant Exotoxin A and its sequence-modified variants.

## 2. Results and Discussion

### 2.1. Analyzed Muteins

**Figure 1:**
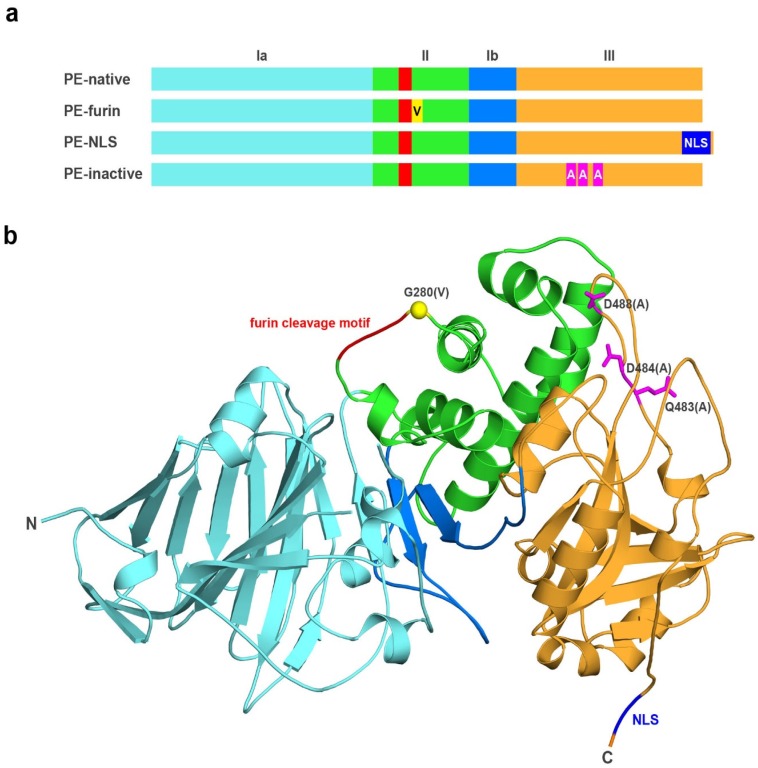
(**a**) Schematic overview; and (**b**) 3D structure of the analyzed toxins. PE comprises of three domains: I (Ia (**light blue**) and Ib (**blue**)); II (**green**); and III (**orange**). The furin cleavage motif (**red**) is located within the domain II. PE-furin possesses a single point mutation (G280V, **yellow**), which renders it resistant to furin cleavage. PE-NLS is characterized by the presence of NLS sequence (RRRKPPPRR, **dark blue**) incorporated at the C-terminus of PE. PE-inactive includes three point mutations (Q483A, D484A and D488A, **magenta**), which abolish its ADP-ribosylation activity.

In this study, we focused on Exotoxin A (PE) and its three PE muteins: PE-NLS, PE-furin and PE-inactive (Figure 1). The newly designed mutein, PE-NLS, has a nuclear localization signal sequence (RRRKPPPRR) inserted at the *C*-terminus (prior to REDLK sequence), which should target the toxin to the nucleus [24]. Importantly, according to the 3D structure, the inserted NLS is not located in the proximity of substrate binding loops and thus the introduced motif should not affect the catalytic activity of the toxin. The PE-furin contains a point mutation G280V in the P1’ position of the furin cleavage site. This mutation makes PE resistant to furin [25,26], as we wanted to verify whether the cleavage by furin is critical for PE activation. The PE-inactive, used as a control, corresponds to a triple mutant (Q483A, D484A and D488A) with negligible ADP-ribosylation activity as reported previously (relative *k*_cat_ value was <0.5% of the wild-type value) [27], while retaining its ability to bind substrates.

### 2.2. Protein Purification

**Figure 2:**
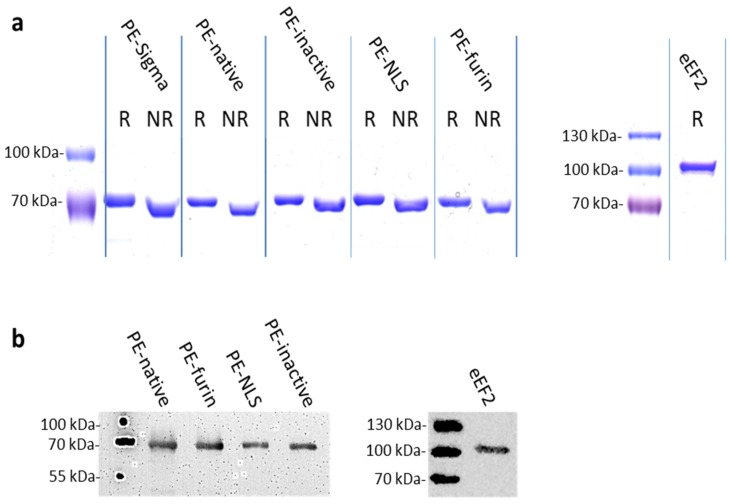
Purified toxins and eEF2: (**a**) Coomassie-stained SDS-PAGE gels with 2 μg of the analyzed proteins under reducing (R) and non-reducing (NR) conditions. PE-Sigma (Sigma Aldrich, St. Louis, MO, USA) was used as a control. (**b**) Western blots with anti-Exotoxin A and anti-eEF2. One hundred nanograms of each protein was used for WB analysis.

Post-translationally modified eEF2 used for ADP-ribosylation assays was purified from *S. cerevisiae* TKY675 strain (Figure 2) using standard purification protocol as described in Materials and Methods.

PE-native was initially expressed in BL21 (DE3) *E. coli* strain and purified in small scale using a protocol analogous to that proposed for eEF2. However, the yield and purity of the protein was very low. The standard protocol was also unsuitable for the analyzed PE muteins, which had a tendency to aggregate during expression. Therefore, we developed a unique and effective method for PE purification (patent pending, PCT/IB2015/058571) by testing various expression and purification conditions. Toxins were overproduced in numerous *E. coli* strains (e.g., BL21(DE3), Tuner(DE3), Rosetta2(DE3), BL21(DE3)CodonPlus-RIL) with different times of induction determined by ODs (OD value ranging from 0.2 to 1.0) and in two growth media (LB and TB) [28]. We focused on lysing the bacterial pellet using various additives like glycerol, NaCl, GSH, EtOH, Triton X100 and urea (0.25 M–8 M) and tested the impact of pH. The combination of urea and basic pH allowed solubilization of the whole pellet with very high abundance of analyzed proteins. We also optimized the protein binding to NiNTA resin, and further purification steps. To remove impurities, we tested size exclusion chromatography of different pore sizes (Sepharose, Superdex and Sephadex; GE Healthcare Life Sciences, Marlborough, MA, USA). We also considered ion exchange chromatography with successful results obtained for Fractogel EMD SE HiCap (M) resin (AEX chromatography). The final procedure includes the usage of low concentration of chaotropic agent (2 M urea) at pH 12.0 to obtain proteins simultaneously from both, the soluble fraction and the inclusion bodies (IB). Prior to purification, the concentration of urea was decreased by simple dilution without any observed aggregation. Mild buffer conditions not only prevent proteins from full unfolding [29] but also seem to have a beneficial effect on the catalytic activity and cytotoxicity of the analyzed toxins (higher activities as compared to the commercially available PE-Sigma, see Table 1 and Table 2). The same positive effect caused by urea was observed by Beattie et al. [30]. To remove the contamination of co-purifying endogenous bacterial proteins (especially the GlmS enzyme (E.C.2.6.1.16) with molecular weight similar to that of the analyzed toxins), we used *E. coli* strain NiCo21 (DE3). CBD-tagged histidine-rich endogenous bacterial proteins were removed using chitin resin [31]. Importantly, the developed protocol seems to be universal for PE-based muteins as it was applied successfully to purify all analyzed toxins (Figure 2). In addition, it ensures reproducible activities of consecutive batches. The purity of the obtained proteins reached 85.6% for PE-NLS, 95.1% for PE-native and 100% for both PE-furin and PE-inactive.

### 2.3. ADP-Ribosylation Activity of Analyzed Toxins

To assess in vitro ADP-ribosylation activity of the analyzed toxins, we adopted protocols for rapid detection and precise quantification of ribosylation, previously used for DT by Bachran et al. [32]. The method for rapid detection is based on Western blot analysis using HRP-conjugated streptavidin, which detects biotin labeled ADP-ribose covalently bound to eEF2. The assay for precise quantification of ADP-ribosylation activity is also based on detection of biotin-labeled ADP-ribose but using color development in a solid-phase assay. Table 1 presents the EC_50_ values obtained for all analyzed toxins.

In the solid-phase assay, the proteins were analyzed in experimentally determined concentration range (from 0.1 pg/μL to 100 ng/μL), which allowed plotting dose–response curves. As expected, no ADP-ribosylation was observed for PE-inactive. However, Western blot revealed that in case of high excess of PE-inactive to its substrate (eEF2), remnant activity can be still detected. We found that, except of PE-inactive, all purified toxins and a commercially available PE (PE-Sigma, obtained from Sigma Aldrich (St. Louis, MO, USA), retain their ADP-ribosylation activity against eEF2 under experimental conditions. We obtained similar values of EC_50_ for PE-native and PE-NLS (Table 1) suggesting that the NLS sequence does not influence ADP-ribosylation activity of the toxin. In the case of PE-furin, the measured activity was slightly higher. The only protein showing significantly lower activity was PE-Sigma, which is obtained by the manufacturer from *Pseudomonas aeruginosa* using a different purification strategy. However, both its ADP-ribosylation activity in vitro and cytotoxicity, was significantly improved upon reconstitution of a lyophilized protein using our newly developed protocol for PE purification (data not shown).

### 2.4. Cytotoxicity of Analyzed Toxins

We measured the cytotoxicity of all analyzed proteins against two different cell lines: fast proliferating A549 (human lung carcinoma, doubling time 22 h) and slowly dividing HepG2 (hepatocellular carcinoma, doubling time 41 h). Table 2 shows the average cytotoxic activities (IC_50_) for toxins, which were assayed at least four times. We found that PE-inactive does not influence the viability of the cells. PE-Sigma was significantly less toxic on both cell lines than PE-native, what is in good agreement with its lower ADP-ribosylation activity. PE-native and PE-Sigma were more toxic on A549 than on HepG2 cells. Surprisingly, unlike for both native toxins, for PE-NLS mutein, we observed comparable cytotoxicity against HepG2 and A549. The vulnerability of slowly dividing cells to PE-NLS was contradictory to our assumption that the toxin targeted to the nucleus would cause less toxic effects. In several experiments (data not shown) on additional cancer cell lines, including MCF-7, SNU-398 and SNU-449, exhibiting different proliferation rate we also observed no difference in cytotoxicity between PE-native and PE-NLS. Therefore, we decided to investigate if PE-NLS is being effectively targeted to the nuclear compartment.

### 2.5. Toxins Accumulation inside the Cell

**Figure 3:**
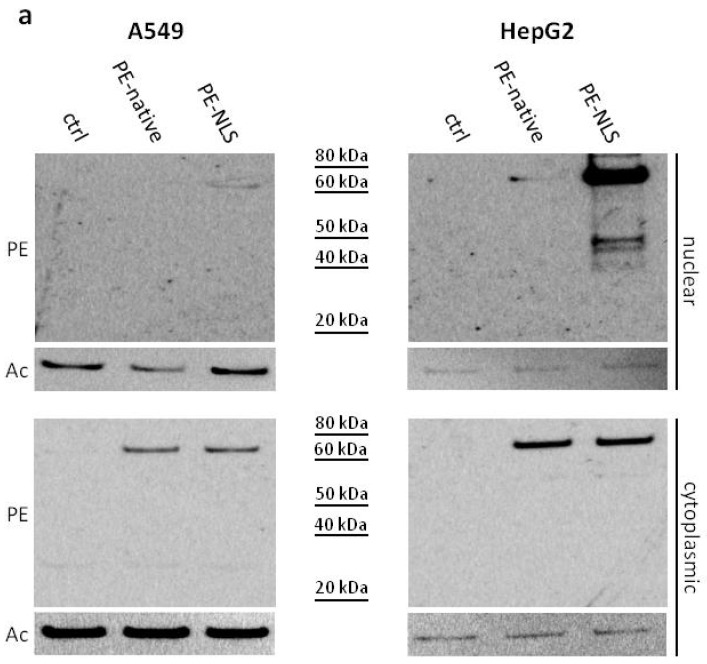

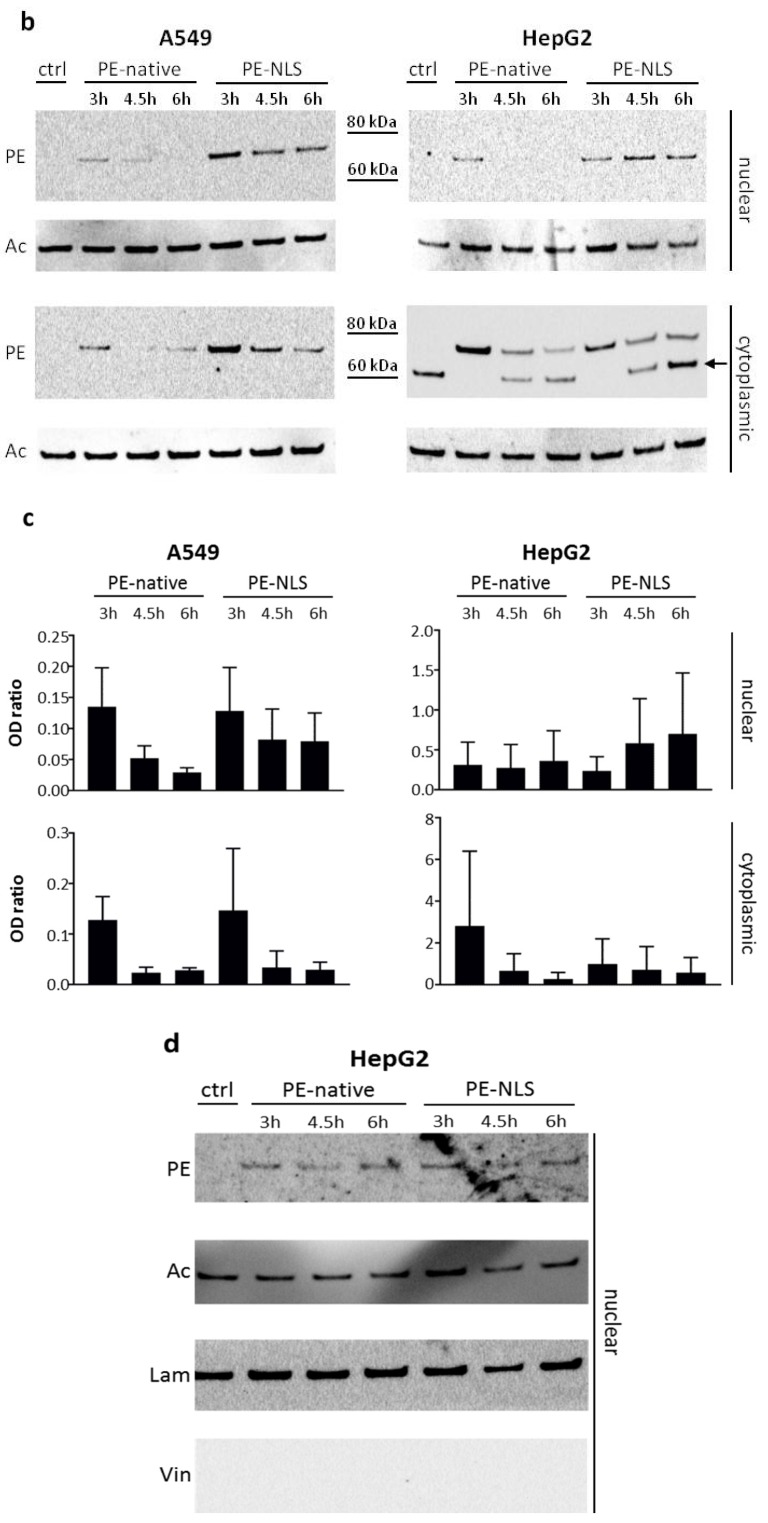
Accumulation of PE-native and PE-NLS in nuclei of A549 and HepG2 cells. (**a**) Representative Western blot of nuclear and cytoplasmic fractions of intoxicated A549 and HepG2 cells. (**b**) Pulse-chase assays. A549 and HepG2 cells intoxicated with PE-native and PE-NLS, were fractionated at different time points to determine the accumulation of toxins in cytoplasmic and nuclear compartments. An arrow indicates a non-specific band. (**c**) Densitometric analysis of blots obtained in pulse-chase experiments. Each point represents mean of OD from three independent experiments normalized to actin. (**d**) Western blot analysis of purity of nuclear fraction obtained from HepG2 cells. Blot was re-probed with antibodies against selected markers of cellular compartments. In all panels, ctrl represents cells not treated with toxin. Blots were probed with anti-PE (PE), anti-actin (Act), anti-lamin (Lam) and anti-vinculin (Vin) antibodies.

Initially, using Western blot, we analyzed the presence of PE-native and PE-NLS in nuclear fractions of A549 and HepG2 cells. We confirmed significant accumulation of PE-NLS in nuclear fractions, especially in HepG2 cells (Figure 3a). Preferential accumulation of PE-NLS in HepG2 cells might be explained by lower doubling time of these cells resulting in a slower process of dilution of the toxin. Interestingly, smaller amounts of PE-native, in both A549 and HepG2 nuclei, were also detected. This is consistent with the recent study by Chaumet et al. on the mechanism of PE transfer inside the cell, showing new endosomal route that transports cell surface receptors to the nucleoplasm [33]. The authors found that PE is located in the nuclear envelope-associated vesicles (NAE) (vesicles associated with nuclear endosomes and derived from early endosomes) and postulated sporadic release of the NAE content into the nucleus. It would explain small amounts of PE-native in the nucleus detected in our study.

Then, we applied pulse-chase assay to monitor accumulation of the toxins in the cytoplasmic and nuclear compartments in time (Figure 3b,c). The prominent accumulation of PE-NLS in HepG2 was unlikely the effect of cross contamination with cytoplasmic proteins as the nuclear to cytoplasmic marker ratio (Figure 3d) suggested high purity of HepG2 derived nuclear fractions. Although unified intoxication procedure was used (the same amount of cells and concentration of toxins in culture medium), concentrations of the analyzed toxins in cytoplasmic fractions of A549 and HepG2 cells differed significantly. Anti-PE antibody has also detected an unknown band in the cytoplasmic fraction of control HepG2 cells (Figure 3b) but this nonspecific band showed clearly different molecular weight than PE-NLS and therefore did not interfere with the assay. The signal for PE-native was around 100 times higher in HepG2 than in A549, while for PE-NLS, the signal in HepG2 was about 10-fold stronger. Consistently, we detected much less LRP receptors on a A549 cell surface (data not shown), which may explain the differences in toxins uptake. In both cell lines, concentrations of toxins were decreasing in time probably due to proteasomal degradation. To verify this hypothesis, we pre-incubated the cells with proteasome inhibitors. Firstly, we determined the vulnerability of both cell lines to proteasome inhibitors MG132 [34] and lactacystin [35] at two concentrations (0.5 μM and 5 μM). Lactacystin showed no effect on viability of neither A549 nor HepG2, while MG132 was toxic to HepG2 (in higher concentration) and had no effect on A549. As intoxication of A549 cells pre-incubated with lactacystin showed no difference in viability, A549 and HepG2 cells were pre-treated with MG132 and lactacystin, respectively, to measure cytotoxicity of PE-native and PE-NLS (Table 3). The viability of A549 and HepG2 decreased two times after intoxication with PE-native. The observed effect is most probably the result of the increased half-live of the toxin due to impaired proteasomal activity. However, the profound effects of proteasome inhibition on different cellular functions including apoptosis and survival [36] might also contribute to the observed effects of proteasome inhibitors on cell response to PE and PE mutein. Interestingly, proteasome inhibition had slightly lower impact on A549 cells intoxicated with PE-NLS (37% decrease of viability), and there was only a very slight increase in vulnerability of lactacystin pre-treated HepG2 cells to PE-NLS intoxication, possibly due to strong accumulation of this mutein in the nucleus. Therefore, we conclude that proteasome degradation might play a role in both, stability of PE (as suggested previously [37]) and its cytotoxicity (similar to DT [38]).

All analyzed toxins were also detected in nuclear fractions from both A549 and HepG2 cells in pulse-chase assay 3 h after intoxication. The amounts of toxins were decreasing in time with the exception of PE-NLS in HepG2, where the prolonged presence of this mutein was observed. These results are consistent with our hypothesis that PE-NLS is preferentially accumulated in nuclei of HepG2 cells in a process involving active transport. The A549 and HepG2 cells were then pre-treated with importazole (selective inhibitor of transport receptor importin-β) [39] followed by determination of their viabilities after intoxication to verify whether importins are involved in the translocation process. For A549 cells, where strong accumulation of PE-NLS in the nucleus was not observed, the viability was not affected, while the viability of HepG2 cells increased (Table 3). This suggests that importins might be engaged in the PE-NLS translocation process. No interaction of unmodified PE with importin β1 (KPNB1, key factor for transport through the nuclear envelope) was detected [33], which suggests that PE cannot migrate between the nucleus and cytoplasm. Consequently, the PE-involved processes in these two compartments seem to be separated. Altogether, it shows that accumulation of PE-NLS in the nucleus increases its cytotoxic activity in HepG2 cells by some unknown mechanism.

**Figure 4:**
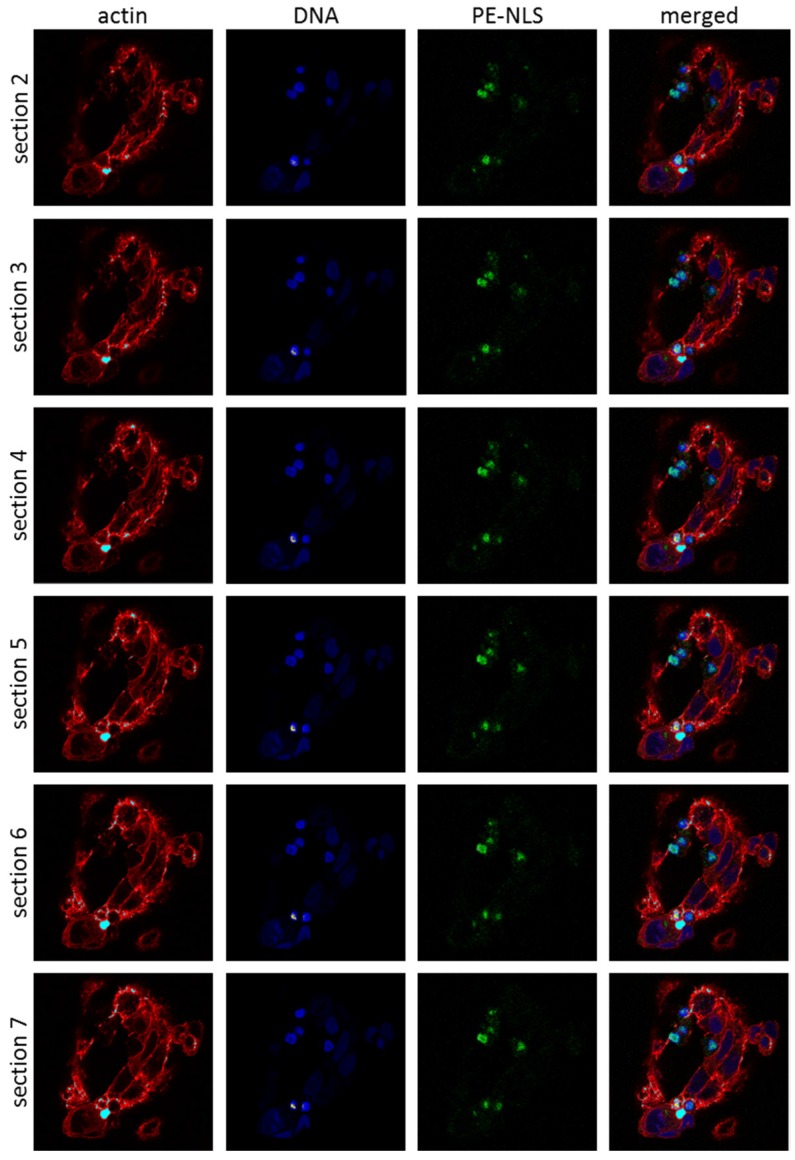
Localization of PE-NLS in HepG2 cells. Cells, intoxicated with fluorescently labeled PE-NLS (**green**), were fixed 3 h after treatment and monitored using confocal microscope. The nuclei were labeled with NucRed Live 647 ReadyProbes Reagent (**blue**), actin was labeled with Alexa Fluor 594 Phalloidin (**red**). The cells were visualized by confocal microscopy at 60× magnification of the objective.

Finally, we decided to directly investigate the intracellular localization of PE-native and PE-NLS within the A549 and HepG2 cells 3 h after intoxication using confocal microscopy. Tetrafluorophenyl was attached directly to the primary amines of the toxins to form stable protein-dye conjugates. PE-native in both cell lines was preferentially distributed within the cytoplasmic compartment, although to a small degree it was also found inside the nuclei (data not shown). In contrast, PE-NLS in HepG2 cells showed a different distribution pattern as it was detected mostly inside and in the proximity of the nuclei (Figure 4). These results are consistent with our previous observations of preferential accumulation of PE-NLS in nuclear fraction obtained from HepG2 cells.

Our hypothesis that NLS sequence, incorporated in PE-NLS, will be recognized by importins and will result in active transport of the mutein to the nucleus seems to be correct but only for HepG2 cells. It remains unclear why PE-NLS is not accumulated inside the nuclei of A549, since A549 cells seem to contain a functional nuclear transport apparatus (importins α/β) [40]. However, lack of nuclear transport of proteins containing NLS sequence in this cell line has already been presented [41]. Similar to our observations, EGFR containing a tripartite NLS (RRRHIVRKRTLRR) was detected in nucleus of HCC827 cells but was barely detectable in nuclear compartment of A549 cells [42,43]. These findings suggest that importins-dependent route in A549 is not efficient as compared to other cell lines.

### 2.6. Furin Cleavage

**Figure 5:**
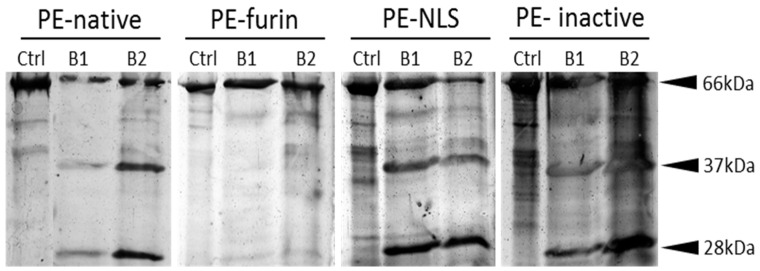
Cleavage by furin. Silver stained SDS-PAGE for cleavage assay in two buffer conditions (B1 and B2). Ctrl represents toxin not mixed with furin protease; and 66 kDa, 37 kDa and 28 kDa correspond to full length, *C*-terminal and *N*-terminal fragments of toxins, respectively.

It was reported that Exotoxin A is activated by furin protease, which cleaves the protein between Arg279 and Gly280 and generates an enzymatically active 37 kDa fragment [8,44]. In all in vivo intracellular localization experiments using Western blotting, we observed only unnicked toxins (bands detected at 66.7 kDa and 67.6 kDa correspond to full-length PE-native and PE-NLS, respectively; e.g., see Figure 3a). It raises a question whether the measured cytotoxicities of PE-native and PE-NLS in the selected cellular models were influenced by the lack of furin digestion. We used a furin resistant mutein (PE-furin) and confirmed that it is not cleaved by furin in any buffer conditions tested in vitro, while PE-native, PE-NLS and PE-inactive underwent proteolysis under the same experimental conditions (Figure 5). Despite the lack of furin processing, PE-furin showed comparable or even slightly higher cytotoxic activity on both cell lines than other toxins. It stays in a good agreement with our results from ADP-ribosylation assay, where PE-furin had the lowest EC_50_ value (Table 1). This indicates that furin cleavage is not crucial for PE activation, at least in the selected cellular models. It is also consistent with recent studies showing that the unnicked toxin is responsible for most of the in vivo ADP-ribosylation of eEF2 [45,46,47].

### 2.7. Deoxyribonclease Activity of PE

**Figure 6:**
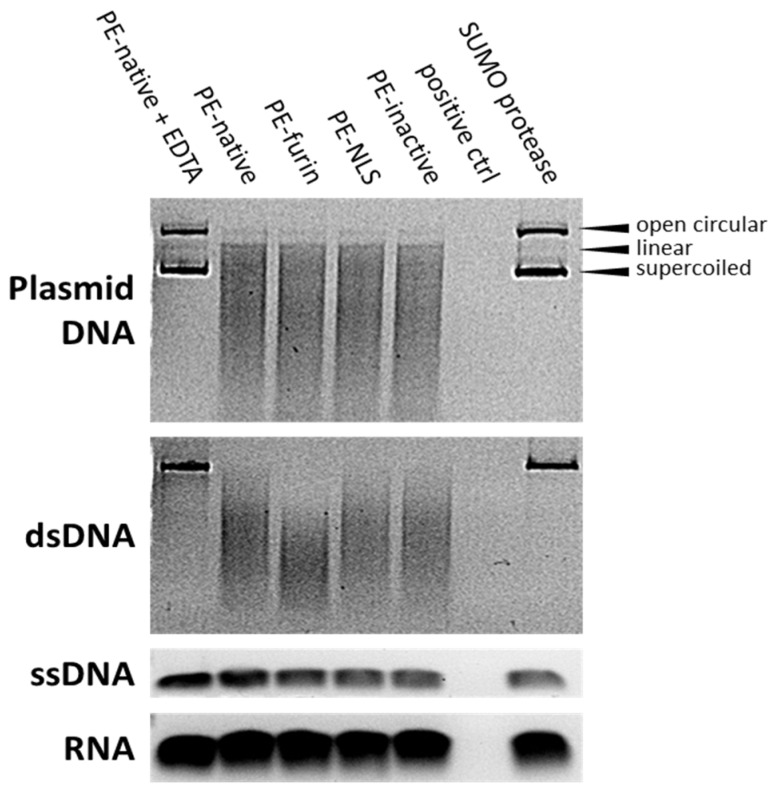
DNA and RNA degradation by analyzed toxins. DNase I and RNase I were used as positive controls for DNA and RNA digestion, respectively. Digestion reactions with addition of PE-native+EDTA and SUMO protease are also shown. All samples were analyzed on 1% agarose gels stained with SYBR Gold (gels with DNA) or SYBR Green II (gel with RNA).

In order to explain the increased cytotoxicity of the nucleus-accumulated PE-NLS, we checked whether this effect might have arisen from the cryptic nuclease activity of the toxin, as a closely related DT has already been reported to cleave DNA in vitro [48,49]. Surprisingly, we found that supercoiled circular plasmid DNA and linear dsDNA mixed with purified toxins is readily degraded in digestion buffer containing Mg^2+^ ions. The analyzed toxins seem to be specific for double stranded DNA (both circular and linear), as no cleavage was observed for ssDNA or RNA (Figure 6). The linear dsDNA digestion can be carried out also in the presence of other than Mg^2+^ divalent cations, i.e., Mn^2+^ and Ni^2+^, and is fully inhibited by addition of EDTA. The optimal temperature for the reaction was oscillating around physiological 37 °C.

**Figure 7:**
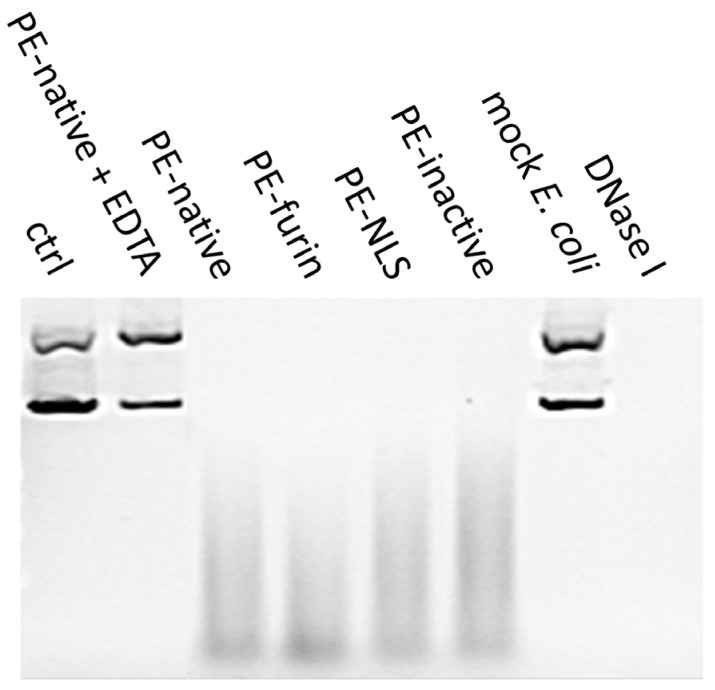
DNA degradation by electroeluted toxins and mock *E. coli* extract. Ctrl represents DNA mixed with TG buffer obtained after electroelution, mock *E. coli* corresponds to proteins with MW from ~55 kDa to 75 kDa. DNaseI was used as a positive control.

To confirm that the nuclease activity was strictly attributed to the toxin and rule out the possible bacterial DNase contamination, the toxins were separated on SDS-PAGE and electroeluted from precisely excised gel bands. Mock preparation of bacterial cells was also separated and the part of the gel representing proteins of the molecular weight comparable to the size of analyzed toxins was excised. All electroeluted samples, except from mock *E. coli* extract, preserved nuclease activity (Figure 7).

### 2.8. DNA Damage in Cells Exposed to PE-NLS

**Figure 8:**
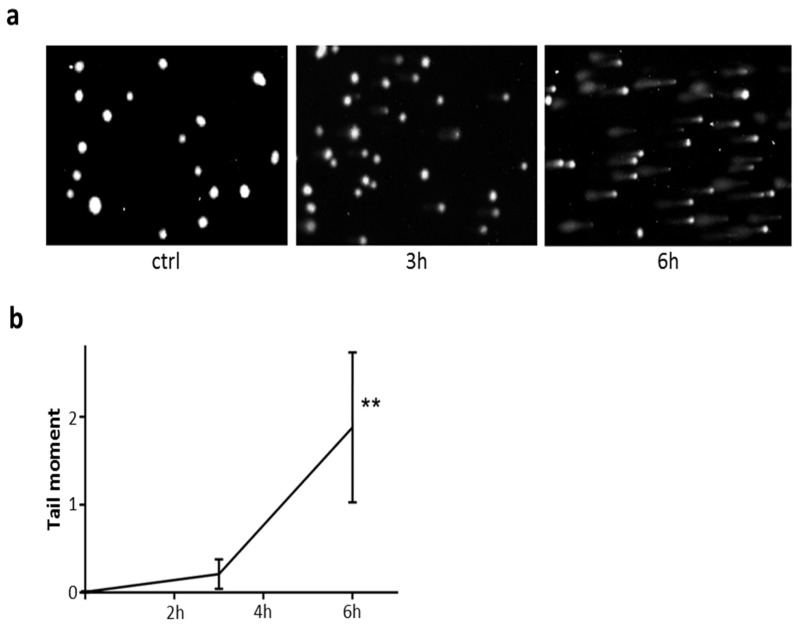
DNA damage in nuclei of cells exposed to PE-NLS. HepG2 cells were exposed to PE-NLS (230 ng/mL) in the presence of pan-caspase inhibitor for 3 h and 6 h, and analyzed by alkaline Comet assay. (**a**) Pictures obtained under fluorescence microscope at 20× magnification. (**b**) Results of quantitative analysis of comets presented as mean ± SEM from 20 randomly selected comets obtained in two independent experiments.

To assess DNA damage in cells exposed to PE-NLS, we employed Comet assays under alkaline conditions. When HepG2 cells were exposed to PE-NLS for 24 h, we observed appearance of multiple comets, suggesting massive DNA damage in the nuclei both in the absence and presence of pan-caspase inhibitor Z-VAD-FMK (data not shown). Next, we investigated the comet formations in earlier time points. As seen in Figure 8a, incubation of HepG2 with PE-NLS in the presence of pan-caspase inhibitor resulted in a time dependent formation of comets. Control performed in the absence of inhibitor showed similar pattern (data not shown). Quantitative image analysis (Figure 8b) confirmed statistically significant increase of average tail moment following 6 h incubation of HepG2 cells with PE-NLS. Thus, accumulation of PE-NLS in the nuclei of HepG2 cells within hours of exposure to this toxin was associated with significant increase in formation of double stranded DNA breaks that was not prevented by inhibition of major pro-apoptotic signaling pathway. These observations are consistent with hypothesis that PE-NLS directly mediates DNA damage in the nuclei.

Altogether, these findings suggest that PE may cause degradation of chromosomal DNA. Interestingly, all analyzed toxins, including PE-inactive, displayed the nuclease activity with similar degradation rates regardless of their ADP-ribosylating efficiency. This may suggest that, similar to DT [50], nuclease activity of PE is independent from its main catalytic activity.

## 3. Conclusions

In this this study, we designed a novel PE mutein by incorporating the NLS sequence at the C-terminus of the toxin [24]. This modification was proposed to target the toxin to the nucleus and separate it from the cellular compartment, where its toxic activity is exerted [51]. We found that NLS sequence does not influence the in vitro ADP-ribosylation activity as suggested by in silico analysis and is highly effective, leading to accumulation of the toxin in the nucleus in one of the cellular models employed in the study. Although we assumed that the toxin enriched with NLS should exhibit lower cytotoxicity because of its separation from its natural substrate eEF2, the results of the cytotoxicity assays were contradictory. Particularly, in HepG2 cells, where the toxin was triggered to preferentially accumulate within the cell nucleus, its toxicity was fully retained. One of the potential explanations is unexpected nuclease activity of PE detected in this study and its potential ability to cleave chromosomal DNA, which could be an alternative intoxication mechanism of the toxin accumulated in the nucleus. Consistently, when nuclear transport apparatus was inhibited by importazole, the viability of HepG2 cells increased. Furthermore, PE-NLS within hours of exposure of cells mediated significant damage of nuclear DNA in a caspase-independent fashion. This suggests that PE is another toxin which has both ADP-ribosylation and nuclease activities, what has been already reported for e.g., Diphtheria toxin from *Corynebacterim diphtheriae* [52] and cytolethal distending toxin from *Salmonella enterica* [53]. However, neither the molecular mechanism of nuclease activity of PE nor the catalytic amino acid residues responsible for this enzymatic activity have been identified yet. Our future investigations will address these issues to provide more insight into this newly detected activity of the toxin.

Additionally, we developed a novel and universal protocol for purification of PE and its muteins. It allowed purification of all analyzed toxins, which retain high biological activity, except of PE inactive triple mutant. Importantly, ADP-ribosylation activity of the toxins obtained using our newly developed method turned out to be higher both, in vitro and in vivo, as compared to commercially available PE and proteins purified using several purification protocols described in the literature (data not shown). In addition, in vitro ADP-ribosylation activities of independent batches of the analyzed PE muteins were comparable. Such reproducibility of the method combined with high activity of the obtained toxins is of great importance for its potential medical applications.

We also analyzed whether furin processing is necessary for toxin activation inside the cell. We detected only full-length toxins in the intoxicated cells and showed that the unnicked toxin (PE-furin) retains undiminished cytotoxicity. This indicates that furin cleavage is not critical for PE activity, at least in some cell types, which is consistent with several previous studies showing lack of correlation between proteolytic processing and PE toxicity [37,54].

The general idea of modifying intracellular fate of PE protein by introducing NLS motif into its amino acid sequence was experimentally confirmed in this study. However, our main assumption that trapping the toxin inside the nucleus would lower the cytotoxicity of PE turned out to be incorrect, possibly due to an unknown alternative toxicity mechanism independent of cytoplasmic localization of the toxin. It suggests that further understanding of detailed mechanisms of PE action is needed for its successful application in cancer targeted therapies. For example, we do not know to what extent the observed nuclease activity participates in PE-mediated cytotoxicity. It is conceivable that by abolishing the nuclease activity (without affecting ADP-ribosylation potential) in combination with incorporation of NLS someone might engineer a PE mutein preferentially active against proliferating cells.

## 4. Materials and Methods

### 4.1. Proteins Expression and Purification

DNA constructs encoding the analyzed toxins were cloned into modified Champion pET SUMO vector using AgeI and HindIII restriction sites. The proteins were overexpressed as *N*-terminal his-tagged SUMO fusions in *Escherichia coli* strain NiCo21(DE3) under the control of inducible T7 promoter activated by 0.5 mM IPTG using TB as growth medium [55]. In each case, pellet was lysed in buffer A (50 mM NaH_2_PO_4_, 300 mM NaCl, 20 mM imidazole, 10% glycerol, pH 12.0) with addition of 2 M urea, 0.5 mM PMSF, 5 mM β-mercaptoethanol, benzonase (5 U/mL), lysozyme (0.1 mg/mL) and Triton X100 (0.05%). Sonicated lysate was diluted 4 times with buffer A at pH 8.0. After centrifugation, crude extract was loaded on chitin resin. Flow through from the chitin column was passed through NiNTA column (Qiagen, Hilden, Germany). The column was washed with buffer containing high salt concentration to remove unspecific binding (50 mM NaH_2_PO_4_, 2 M NaCl, 20 mM imidazole, 10% glycerol, pH 8.0). Toxin was eluted in gradient elution mode with buffer 50 mM NaH_2_PO_4_, 300 mM NaCl, 500 mM imidazole, 10% glycerol, pH 8.0. SUMO protease was added to the eluate (to remove his-tagged SUMO) and left at 4 °C overnight. The protein was loaded on size exclusion column (HiLoad Superdex 200, GE Healthcare Life Sciences, Marlborough, MA, USA) equilibrated with phosphate buffer saline amended with 10% glycerol, pH 7.4. The collected fractions were concentrated using Vivaspin Turbo. Protein concentrations were measured using Bradford protein assay (BioRad, Hercules, CA, USA). The proteins were subsequently analyzed by Western blot and using SDS-PAGE gels to identify their purity.

His-tagged eEF2 was expressed in *Saccharomyces cerevisiae* strain TKY675 in 12 L of yeast extract peptone dextrose medium for 22 h at 30 °C. Pellets obtained after centrifugation were frozen in liquid nitrogen and rapidly disrupted in stainless-steel mill. The disrupted cells were suspended in 1 L of lysis buffer (50 mM NaH*_2_*PO_4_, 300 mM NaCl, 20 mM imidazole, 10% glycerol, pH 8.0). The lysate was sonicated, centrifuged and filtered, and then loaded on a NiNTA column. The protein was further purified and analyzed as described above.

### 4.2. ADP-Ribosylation Assays

#### 4.2.1. Western Blotting with Anti-Biotin

1 μg of toxin was mixed with 600 ng of eEF2, 0.5 μL of biotinylated NAD+ (250 μM, Trevigen, Gaithersburg, MD, USA) in a total volume of 24 μL reaction buffer containing 50 mM Tris, 1 mM EDTA, 1 mM DTT, 500 mM NaCl, pH 7.6. The reaction mix was incubated for 1 h at 37 °C. The samples were separated by electrophoresis and transferred to PVDF membrane. The detection was performed using HRP-conjugated streptavidin (Sigma Aldrich, St. Louis, MO, USA) at concentration 1:7000.

#### 4.2.2. Solid-Phase Assay

U16 MaxiSorp plate (Nunc A/S, Roskilde, Denmark) was precoated with goat anti-mouse antibody (GAM) (Agilent Technologies, Santa Clara, CA, USA) .Antibody was diluted 1:100 in PBS and 100 μL of the mixture was placed in each well for 2 h. To avoid cross-contamination, every second column of wells was coated. The wells were washed three times with fresh PBST (PBS with 0.05% Tween 20), soaked for 30 min with the same buffer and washed again. GAM precoated wells were incubated overnight with 100 μL of PBS with penta-his antibody (Qiagen, Hilden, Germany) (final concentration of antibody 5 μg/mL), and then washed with PBST.

100 μL reaction mix contained 1.2 μg of His-tagged eEF2, 5 μM of biotinylated NAD+, 50 mM Tris (pH 7.6), 10 μL BSA (3 mg/mL), 10 mM l-arginine, 0.5 mM NaCl, 1 mM EDTA, 1 mM DTT and 1 μL of toxin (concentration ranging from 0.1 ng/mL to 100 μg/mL). The reaction was incubated for 2 h at 37 °C and transferred to freshly washed coated wells. Each toxin was tested in triplicate on a separate plate with PE-inactive as a control and background indication.

The reaction mixture was incubated on a 96-well plate for 2 h at constant 21 °C without shaking. Precoated wells were washed and soaked with PBST between all steps. After incubation and washing, the wells were filled with blocking solution (50 mg/mL dry milk powder in 200 μL PBS) for 30 min at 21 °C, followed by 30 min at 37 °C. After incubation with HRP-conjugated streptavidin (Sigma Aldrich) for 30 min at 21 °C (1 μg/mL of antibody in 100 μL PBS containing 100 μL/mL FBS and 30 mg/mL BSA), the wells were washed again and finally incubated with 100 μL of 3,3’,5,5′-tetramethylobenzidine (0.1 mg/mL TMB resuspended in 0.05 M citric-phosphate buffer, 0.01% H_2_O_2_, pH 5.0) for color development. The reaction was stopped after 30 min by addition of 50 μL of 2 M H_2_SO_4_. The absorbance in each well was measured at 450 nm and 490 nm, using Enspire microplate reader (Perkin Elmer, Waltham, MA, USA). The EC_50_ values were calculated from dose–response curves using GraphPad Prism 5 software (version 5.0 for Windows, GraphPad Software, Inc., San Diego, CA, USA, 2010). 

### 4.3. Cytotoxicity Measurements

The HepG2 cells and A549 cells were obtained from ATCC (Manassas, VA, USA) and were maintained under standard conditions in Dulbecco’s Modified Eagle’s Medium supplemented with 10% fetal bovine serum, 100 U/mL penicillin and 100 μg/mL streptomycin at 37 °C in a humidified atmosphere of 5% CO_2_. The passage number range for both cell lines was maintained between 20 and 25.

To estimate the cytotoxicity of the investigated toxins, the neutral red uptake assay was used [56]. HepG2 and A549 cell lines were seeded into 96-well plates at a density of 1.5 × 10^4^ cells per well. After 24 h, the cells were treated with increasing concentrations of the toxins and incubated for another 48 h. Cells treated with CHX (final concentration 20 μg/mL) and SDS (final concentration 200 μg/mL) were used as additional controls for sensitivity of cells to inhibition of protein synthesis and to standard cell membrane damaging cytotoxic agent, respectively. After incubation, the medium was removed and the cells were washed with cold PBS. The washed cells were incubated with 50 μg/mL neutral red in HBSS for 3 h. Following incubation, the neutral red solution was removed, the cells were washed with PBS and the cell-bound dye was extracted using a solution containing 50% ethanol and 1% acetic acid by gentle shaking for 10 min. Absorbance at 550 nm was determined using a Sunrise microplate reader (Tecan, Männedorf, Switzerland). The IC_50_ values were calculated based on linear dose–response curves using GraphPad Prism 5 software (version 5.0 for Windows, GraphPad Software, Inc., San Diego, CA, USA, 2010).

### 4.4. Viability Measurements

HepG2 and A549 cells were seeded into 96-well plates at a density of 1.5 × 10^4^ cells per well, and after 24 h cells were preincubated for 1 h with the following inhibitors: MG132, lactacystin and importazole (all inhibitors from Sigma Aldrich). The inhibitors were added at a final concentration of 5 μM. Medium was not removed after incubation and cells were supplemented with pure medium (control cells) or treated with selected toxins at concentrations equal to IC_50_ values for another 48 h. After that time, the incubation medium was removed, and the viability was assessed as described above for cytotoxicity measurements.

### 4.5. Intracellular Localization Studies

#### 4.5.1. Western Blotting with Anti-Exotoxin A

For Western blotting, cells were pretreated with toxins (PE-native and PE-NLS) for 3 h, 4.5 h and 6 h, and then cytoplasmic and nuclear fractions were prepared: cells were lysed for 10 min at room temperature in a buffer containing 0.4% IGEPAL CA-630, 10 mM HEPES (pH 7.9), 10 mM KCl, 0.1 mM EDTA, and 1× Roche protease inhibitor mixture. Lysates were centrifuged at 15,000× *g* for 3 min at 4 °C. Supernatants (cytosolic fractions) were collected and used directly in Western blots or stored at −80 °C. The pellets were suspended in a buffer containing 20 mM HEPES (pH 7.9), 0.4 M NaCl, 1 mM EDTA, 10% glycerol and 1 × Roche protease inhibitor mixture; shaken vigorously for 2 h at 4 °C; and centrifuged at 15,000× *g* for 5 min at 4 °C. The resulting supernatants (nuclear extracts) were collected and used directly in Western blots or stored at −80 °C. Protein concentrations were determined using Pierce™ BCA Protein Assay Kit (Thermo Fisher Scientific, Waltham, MA, USA).

The fractions were separated using electrophoresis and transferred to a membrane using the iBlot Dry Blotting System (Life Technologies, Carlsbad, CA, USA). After overnight incubation with blocking agent (SuperBlock (TBS) Blocking Buffer, Thermo Fisher Scientific), the membrane was incubated with anti-*Pseudomonas* Exotoxin A (Sigma Aldrich, St. Louis, MO, USA) for 1 h (1:5000). The primary antibody was detected with goat anti-mouse peroxidase conjugated secondary antibody (Agilent Technologies, Santa Clara, CA, USA) (1:2000) and visualized using SuperSignal West Pico Chemiluminescent Substrate (Thermo Fisher Scientific, Waltham, MA, USA) Quantification of individual signals was normalized to the level of α-actin (SC-1616 HRP antibody, Santa Cruz, Santa Cruz, CA, USA). 

#### 4.5.2. Confocal Microscopy

For confocal microscopy, cells were pretreated for 3 h with toxins (PE-native and PE-NLS) labeled with Alexa Fluor 488 Dye (Thermo Fisher Scientific) according to manufacturer protocol. The cells were fixed (4% formaldehyde, for 10 min) and stained with fluorescent dyes (NucRed Live 647 ReadyProbes Reagent for nuclei visualization and Alexa Fluor 594 Phalloidin for actin visualization; Thermo Fisher Scientific) in standard conditions, and visualized under a Nikon C1 confocal microscope.

### 4.6. Furin Digestion Assay

The efficacy of furin cleavage was tested in two buffers: B1 (10 mM HEPES, 0.05% Triton X100, 1 mM CaCl_2_, 1 mM β-mercaptoethanol, pH 7.5) and B2 (0.05 mM citric acid, 100 mM NaH_2_PO_4_, 1 mM CaCl_2_, 1 mM β-mercaptoethanol, pH 5.5). Two thousand five hundred nanograms of each toxin was diluted to 100 μL in B1 or B2 and incubated for 24 h at 37 °C with 2 U of recombinant furin (NEB). The cleavage efficiency was assessed using both, silver stained SDS-PAGE and Western blot.

### 4.7. Agarose Gel Nuclease Assays

#### 4.7.1. DNA/RNA Degradation

The analyzed toxins (2 μg) were mixed with nucleotide substrates: 200 ng of dsDNA (Xho I—linearized pBSK plasmid treated with alkaline phosphatase and circular pBSK), 20 ng of ssDNA (synthetic oligonucleotide GACTGGAGCACGAGGACACTGACATGGACTGAAGGAGTAGAAA) and 10 ng of RNA (synthetic oligonucleotide CGACUGGAGCACGAGGACACUG) in 20 μL of digestion buffer (10 mM Tris, 10 mM MgCl_2_, 1 mM CaCl_2_, pH 7.5). Reaction was incubated for 16 h at 37 °C and terminated by mixing with gel loading buffer (6× DNA Loading Dye, Thermo Fisher Scientific). Digestion reactions were also prepared with addition of 20 mM EDTA simultaneously with PE-native or 2 μg of SUMO protease. For positive controls, 2 U of DNase I (Thermo Fisher Scientific, Waltham, MA, USA ) and 2 U of RNase I (Thermo Scientific) were used for DNA and RNA degradation, respectively. Samples were analyzed on 1% agarose gels stained with SYBR Gold (DNA) or SYBR Green II (RNA) and visualized under UV light.

Nuclease assays with 2 μg of PE-native toxin and dsDNA fragment were performed in various conditions: (i) using different cations—MgSO_4_, MnCl_2_, NaCl, CaCl_2_, ZnCl_2_, FeCl_3_, NiSO_4_, CoCl_2_, KCl, and MgCl_2_ salts were added to the Tris buffer (10 mM salt, 10 mM Tris, pH 7.5); and (ii) in a broad temperature range (4 °C–54 °C) in Tris buffer (2.5 mM MgSO_4_, 10 mM Tris, pH 7.5). All samples were incubated for 24 h at 37 °C (except for ii) and analyzed on 1% agarose gels stained with Midorii Green.

#### 4.7.2. Electroelution

Analyzed toxins (10 μg) and 60 μL of total lysate of NiCo21 (DE3) were mixed with 6× Laemmli buffer without heating and separated on 10% SDS-PAGE. The gel bands of interest (toxins), visualized by Ponceau S, were excised and placed in dialysis tubes with 250 μL of TG buffer. In addition, fragment of the gel containing proteins ranging from ~55 kDa to 75 kDa were excised from the separated mock *E. coli* extract. Tubes were placed in horizontal electrophoresis chamber with constant 10 mV for 24 h [57]. Thirty microliters of the eluted and filtered toxins were mixed with 200 ng of circular DNA (circular pBSK plasmid) in buffer 10 mM Tris, 10 mM MgCl_2_, 1 mM CaCl_2_, pH 7.5 (total volume 50 μL). Digestion reaction was also mixed with 30 μL of TG used for electroelution or PE-native with addition of 20 mM EDTA**.** For positive control, 2 U of DNAse I (Thermo Scientific) was used. Samples were incubated in 37 °C and analyzed after 96 h on 1% agarose gels stained with SYBR Gold.

### 4.8. Alkaline Comet Assay

Assessment of DNA damage was carried out using the alkaline CometAssay^®^ Kit (Trevigen, Gaithersburg, MD, USA) according to the manufacturer protocol. Briefly, HepG2 cells (1 × 10^5^ cells per well) were exposed to PE-NLS (230 ng/mL) in the presence of Pan-Caspase Inhibitor Z-VAD-FMK (50 μM) in 24-well plates for 3 h and 6 h at 37 °C. The H_2_O_2_ (50 μM) was used as a positive control. After washing twice with PBS, the cells were suspended in 1 mL of PBS and scraped. The cell suspension was centrifuged at 3000 rpm for 5 min and the pellet was suspended in 100 μL of PBS. After that, 1 × 10^5^ cells were combined with molten LMAgarose (at 37 °C) at a ratio of 1:10 (*v*/*v*), placed (50 μL) on CometSlide and kept for 10 min at 4 °C. The cells were lysed in a lysing solution for 60 min. After the lysis, the slides were placed in alkaline solution for 20 min to allow DNA unwinding, and then electrophoresed for 30 min with 21 V. All preparative steps were conducted in dark to prevent secondary DNA damage. Cells were stained with 100 μL of diluted (1:10,000 in TE Buffer, pH 7.5) SYBR Green I for 30 min (room temperature) in the dark. The slides were analyzed at 20× magnification using a fluorescence microscope (Olympus BH2-RFCA with Hamamatsu ORCAII BT-1024 camera (Hamamatsu, Hamamatsu City, Japan). Comets were quantitatively analyzed using Comet Assay Software Project casp-1.2.2 (University of Wroclaw, Wroclaw, Poland). Each treatment was carried out in duplicate and 20 randomly selected comets from two microscope slides were analyzed.

### 4.9. Statistical Analysis

IC_50_ were calculated using nonlinear regression of log (concentration of toxin) vs. normalized response using variable slope model. In analysis of statistical significance of differences in dose response to PE and PE muteins, the area under the curve for dose response curves obtained in individual experiments were determined and used in multiple *t*-test comparison (with Holmak–Sidak adjustment). One way unpaired ANOVA followed by Dunnett’s multiple comparisons test was employed in other analyses. All statistical analyses were performed using GraphPad Prism 7.02 software (version 7.02 for Windows, GraphPad Software, Inc., San Diego, CA, USA, 2016). 

## Figures and Tables

**Table 1:** The EC_50_ values for the analyzed toxins in the ADP-ribosylation assay. The 2-h EC_50_ values were determined in triplicates in solid-phase assay with *S. cerevisiae* eEF2 as a substrate. PE-inactive showed no ADP-ribosylation activity. PE-Sigma (Sigma Aldrich, St. Louis, MO, USA) was used as a control protein.

Toxin	EC_50_ ± SD (μg/mL)
PE-native	0.54 ± 0.01
PE-inactive	-
PE-NLS	0.57 ± 0.19
PE-furin	0.40 ± 0.08
PE-Sigma	3.00 ± 0.40

**Table 2:** Cytotoxicity of PE and its muteins on A549 and HepG2 cells. A549 and HepG2 cells were incubated with PE-native, PE-NLS, PE-furin and PE-Sigma (control protein) for 48 h. PE-inactive showed no cytotoxicity on both cell lines. The IC_50_ values are representative of four experiments for each protein. * Statistically significant difference at *p* < 0.05.

Toxin	IC_50_ ± SD (ng/mL)	
	A549	HepG2
PE-native	12 ± 4	22 ± 4 *
PE-inactive	-	-
PE-NLS	17 ± 6	13 ± 4
PE-furin	10 ± 1	13 ± 2
PE-Sigma	425 ± 65	3197 ± 1030 *

**Table 3:** Viability of A549 and HepG2 cells treated with proteasome and transport receptor importin-β inhibitors, and intoxicated with PE-native and PE-NLS. A549 cells were pre-incubated with MG132 (proteasome inhibitor) or importazole (transport receptor importin-β inhibitor), while HepG2 cells were pre-incubated with lactacystin (proteasome inhibitor) or importazole. Both cell types were then intoxicated with PE-native or PE-NLS. Viability was assessed after 48 h. Ctrl represents cells treated only with inhibitors. The values represent means ± SEM of four experiments. * Statistically significant difference at *p* < 0.05; ** statistically significant difference at *p* < 0.01.

Toxin	Viability (%)					
	A549	A549 + MG132	A549 + Importazole	HepG2	HepG2 + Lactacystin	HepG2 + Importazole
PE-native	49.1 ± 2.8	24.7 ± 2.7 *	53.3 ± 6.2	49.7 ± 2.0	27.9 ± 2.3 *	54.3 ± 1.0
PE-NLS	52.2 ± 2.7	32.6 ± 4.9 *	56.2 ± 3.8	52.9 ± 3.6	42.5 ± 3.1 *	68.7 ± 1.0 **
Ctrl	-	82.6 ± 0.5	122.5 ± 3.3	-	100.3 ± 0.8	100.1 ± 4.3
